# Automatic Generation of Number Series Reasoning Items of High Difficulty

**DOI:** 10.3389/fpsyg.2019.00884

**Published:** 2019-04-24

**Authors:** Luning Sun, Yanan Liu, Fang Luo

**Affiliations:** ^1^The Psychometrics Centre, University of Cambridge, Cambridge, United Kingdom; ^2^Beijing Key Laboratory of Applied Experimental Psychology, Faculty of Psychology, Beijing Normal University, Beijing, China

**Keywords:** automatic item generation, number series, high difficulty, LLTM, stimulus features

## Abstract

Number series reasoning items have been frequently used in educational assessment. This study reviewed the previous literature investigating features relating to item difficulty and developed a new automatic generator for number series reasoning items. Ninety-two items were generated and administered to 466 students. The results showed that the test achieved acceptable reliability. Items requiring two arithmetic operations were of particularly high difficulty. All stimulus features implemented in the automatic item generator proved to be significant predictors of item difficulty, explaining 77% of the total variance. This suggests that the automatic number series reasoning item generator was capable of generating items of considerably predictable difficulty. More importantly, the resulting items covered a wide range of difficulty levels, meeting the particular need for items of high difficulty.

## Introduction

In educational assessment, reasoning tests have been extensively employed to evaluate fluid intelligence. The number series completion problem is one of the most frequently used reasoning tasks (Horn and Noll, 1997). However, despite its wide application, there are known challenges in the development of number series reasoning items, including the significant time and effort required to generate items of high quality (Rudner, 2010). Particularly, items that are developed manually are usually limited by the ability level of their developers, and thus often found to be similar and to have low psychometric properties, covering only a narrow range of difficulty levels (Hornke and Habon, 1986; Wainer, 2002). Since the 1980s, researchers have pioneered research on automatic item generation, aiming at relieving the burden on item developers (Irvine and Kyllonen, 2002). This new approach is considered a handshake between cognitive science and psychometrics, enabling effective control of item difficulty based on cognitive processes involved in assessment, and leading to improved test development and educational measurement.

### Automatic Item Generation (AIG)

Automatic item generation (AIG) relies on computer programs to automatically generate items of predictable difficulty according to specific requests made by test developers (Embretson, 2002; Embretson and Yang, 2006). It usually consists of two phases. In the first phase, test development experts propose item stimulus features that affect the cognitive processes involved in assessment. Subsequently, item models are constructed on the basis of these features. In the second phase, items are derived from the item models through feature manipulation carried out by computer programs (Gierl and Lai, 2012).

There are two common approaches to AIG, the item model approach and the cognitive design system approach (Embretson and Yang, 2006). In the item model approach, item models are pre-specified based on existing tests that have been empirically shown to be reliable and valid. New items are constructed by replacing features that are not relevant to the problem solving process, such as the names of objects and specific numbers. The Educational Testing Service (ETS) has reported that they have successfully employed this approach to enlarge their item pool (e.g., Bejar, 1996, 2002; Sinharay and Johnson, 2005). However, researchers have argued that items created by the item model approach do not necessarily possess comparable psychometric properties (Arendasy et al., 2007). It is also possible for test takers to learn item models through extensive practice, resulting in an invalidated item pool (Morley et al., 2004).

Compared to the practically simple item model approach, the cognitive design system approach takes a different and more complex route. This approach strives to bridge cognitive psychology with psychometrics by systematically modifying stimulus features when generating new items. Cognitive psychology focuses on the mental processes of problem solving, whereas psychometrics is concerned with the quality of the measurement tools. The cognitive design system aims at understanding the cognitive processes that test takers employ to complete a test and identify the stimulus features that affect these cognitive processes as well as their influences on item properties and the overall test results (Embretson, 2004; Embretson and Yang, 2006; Gorin, 2006). When new items are generated, their difficulty levels can be modeled and predicted on the basis of these features. This approach also allows for extensive combinations of effective and irrelevant features, resulting in more diversified items than is possible with the item model approach.

The cognitive design system approach uses the Linear Logistic Test Model (LLTM) to predict item difficulty (Fischer, 1973; Freund et al., 2008; Holling et al., 2009). The LLTM is a linear combination of multiple stimulus feature difficulties,

$$P(x_{\text{ij}}=1)=\frac{\exp\left[{\text{θ}}_{\text{j}}-\left(\sum_{\text{m}}{\text{η}}_{\text{m}}q_{\text{im}}+d\right)\right]}{1+\exp\left[{\text{θ}}_{\text{j}}-\left(\sum_{\text{m}}{\text{η}}_{\text{m}}q_{\text{im}}+d\right)\right]}$$

wherein *P(x*_ij_ = *1)* is the probability of participant *j* correctly answering item *i*; θ_j_ is the ability parameter of participant *j*; η_m_ is the difficulty of stimulus feature *m* of item *i*; *q*_im_ is the weight of the difficulty of stimulus feature *m* of item *i*; and *d* is a normalization constant.

The LLTM enables the difficulty parameters of individual stimulus features to be predicted. Item difficulty, which is a linear combination of the difficulties of stimulus features involved in the item, can also be predicted. If the item difficulties predicted by the LLTM are highly correlated with those derived from the Rasch model (Rasch, 1960) or an Item Response Theory model (which empirically estimates item difficulty from participants’ response data), we can assume good performance of the AIG system. In another words, a substantial proportion of the variance in item difficulties can be explained by the stimulus features modeled in the LLTM, suggesting that the difficulty levels of new items are primarily influenced by model stimulus features rather than unspecified or irrelevant features. Consequently, a large number of new items with predictable difficulty levels can be conveniently generated.

### Stimulus Features of Number Series Reasoning Items

The number series completion problem, as an important type of reasoning tasks, aims at assessing test takers’ ability to detect patterns within number series (Klauer, 1996). Simon and Kotovsky (1963); (Kotovsky and Simon, 1973) proposed a cognitive framework for the letter series completion problem, which was later extended to number series by Holzman et al. (1983). The framework includes four components: relation detection, discovery of periodicity, pattern description, and extrapolation. First, during relation detection, test takers attempt to make a hypothesis about the relations among the numbers in the series. After they discover the periodicity, they identify either a single number series or several intertwined series. Subsequently, they process the initial elements and the relations between adjacent elements in their working memory in order to depict the pattern. Finally, the missing number is derived by extrapolation based on the hypothesis that was previously confirmed.

Based on this cognitive framework, Holzman et al. (1983) suggested a range of stimulus features involved in individual phases, including working memory demands, period length, pattern description length, relational complexity, category of arithmetic operation, and string length. These features are not independent. For example, working memory demands are related to relational complexity; the more complex the relation, the greater the demand on working memory. Similarly, Arendasy and Sommer (2012) reported four stimulus features that affect the difficulty of number series reasoning items: periodicity, rule span, number of rules, and rule complexity. Periodicity refers to the number of periods to be discovered. Rule span denotes the number of arithmetic operations per linked elements. Number of rules specifies the number of different rules to be inferred. Rule complexity is concerned with relational complexity, particularly with respect to different categories of arithmetic operation. In addition to the commonly used ideas of arithmetic and geometric progression, Verguts et al. (2002) drew on four new rules in their number series construction: addition, Fibonacci, interpolation, and multiplication.

### Automatic Generation of Number Series Reasoning Items of High Difficulty

There is a high demand for number series reasoning items, as this item type has been frequently employed in aptitude tests and other high-stake examinations. AIG offers considerable benefits in improving item generation (Huff et al., 2013). It not only lowers the item exposure rate, reducing concerns around test security, but it is also more fair and produces more accurate scores, as parallel items can be produced more easily. More importantly, new items can be created on the basis of item models rather than individual items, which significantly enhances efficiency and reduces the costs incurred during item development and pilot testing.

Automatic item generation has been applied to the development of a variety of item types. For example, Arendasy and colleagues successfully employed AIG to develop algebra verbal reasoning items (Arendasy et al., 2006, 2007), matrices reasoning items (Arendasy and Sommer, 2005), word fluency items (Arendasy et al., 2012), and mental rotation items (Arendasy and Sommer, 2010). AIG has also been used to generate abstract reasoning items (Embretson, 2002), figural analogy items (Blum et al., 2016), Latin square tasks (Zeuch et al., 2011), and probability verbal reasoning items (Holling et al., 2009). In particular, Arendasy and Sommer (2012) developed an automatic number series item generator, with which they introduced four item features (as described above) and created 30 item models.

There is an urgent need for difficult mathematical questions in China, as students in many regions of the country exhibit high mathematical aptitude. For instance, in the PISA 2012 test, students from Shanghai (the only region in mainland China that participated) achieved an average score of 613 on Mathematics – 117 points higher than the average score across OECD countries and ranking them 1st in the world (Kelly et al., 2013). When the Wechsler Intelligence Scales were adapted in China, the most difficult items were considered only moderately difficult, and more challenging items had to be developed (Zhang, 2009; Cui et al., 2017). Most existing number series reasoning items are not sufficiently difficult for these students; hence, we often find ceiling effects and poor discrimination among high achievers. Such items are not applicable to assessment scenarios in China.

The current study aimed at identifying the appropriate stimulus features of number series and designing an automatic item generator capable of creating number series reasoning items of high difficulty. Based on 18 item models, 4 item sets were generated and administered. Subsequently, an item difficulty prediction model was constructed and evaluated with the empirical data, in order to examine the reliability and validity of the item generator.

## Materials and Methods

### Development of the Automatic Item Generator

Focusing on arithmetic operation(s) and number elements, which are essential to number series, the current study proposed three classes of stimulus features: type of number element, type of arithmetic operation, and number of arithmetic operations. Specific features (i.e., rules) were carefully designed so as to broaden the difficulty range. We introduced three types of number elements: integers, fractions, and irrational numbers (square roots of integers such as $\sqrt{2}$). Fractions and irrational numbers have rarely been used in number series; hence, we expected that they would increase the level of difficulty. We used four types of arithmetic operations: arithmetic sequences (with a constant increment between consecutive numbers; e.g., 2, 4, 6, 8, 10); geometric sequences (with a constant ratio between consecutive numbers; e.g., 2, 4, 8, 16, 32); addition sequences (with each number being the sum of the two immediately preceding numbers, except for the first two numbers, similar to a Fibonacci sequence; e.g., 2, 3, 5, 8, 13); and multiplication sequences (with each number being the product of the two immediately preceding numbers, except for the first two numbers; e.g., 2, 3, 6, 18, 108). Each number series required either one or two steps of arithmetic operation. Combinations of these stimulus features gave rise to a total of 18 item models.

As it is not easy to add irrational numbers, we did not include such numbers in our arithmetic sequences or addition sequences. Ten item models required only one step of arithmetic operation to deduce the answer (see Table 1).

**Table 1:** Description of item models requiring only one step of arithmetic operation.

Item model	Type of number element	Type of arithmetic operation	Model description^∗^	Example
1	Integer	Arithmetic sequence	a_n+1_ = a_n_+ k	3, 6, 9, 12, 15
2	Integer	Geometric sequence	a_n+1_ = a_n_× k	3, 6, 12, 24, 48
3	Integer	Addition sequence	a_n+2_ = a_n+1_+ a_n_	3, 3, 6, 9, 15
4	Integer	Multiplication sequence	a_n+2_ = a_n+1_× a_n_	3, 3, 9, 27, 243
5	Fraction	Arithmetic sequence	a_n+1_ = a_n_+ k	1/2, 1, 3/2, 2, 5/2
6	Fraction	Geometric sequence	a_n+1_ = a_n_× k	1/2, 1/4, 1/8, 1/16, 1/32
7	Fraction	Addition sequence	a_n+2_ = a_n+1_+ a_n_	1/3, 2/3, 1, 5/3, 8/3
8	Fraction	Multiplication sequence	a_n+2_ = a_n+1_× a_n_	1/3, 2/3, 2/9, 4/27, 8/243
9	Square root	Geometric sequence	a_n+1_ = a_n_× k	$\sqrt{3}$, 3, 3$\sqrt{3}$, 9, 9$\sqrt{3}$
10	Square root	Multiplication sequence	a_n+2_ = a_n+1_× a_n_	$\sqrt{2}$, $\sqrt{3}$, $\sqrt{6}$, 3$\sqrt{2}$, 6$\sqrt{3}$

*^∗^k is a constant. In the current study, k was an integer no larger than 5. a_n_, a_n+1_, and a_n+2_ were consecutive elements in the number series.*

When confronting items requiring two steps of arithmetic operation, test takers needed to first calculate the increments or ratios between consecutive numbers; doing so would form another number series (i.e., a derivative series, which was shorter than the primary series by one number). The same four types of arithmetic operation as used in items requiring one arithmetic operation were employed in the derivative series. Only integers were used in such series. In total, eight item models requiring two steps of arithmetic operation were created (see Table 2).

**Table 2:** Description of item models that require two steps of arithmetic operations using only integers.

Item model	Type of arithmetic operation at Step 1	Type of arithmetic operation at Step 2	Model description^∗^	Example
11	Arithmetic sequence	Arithmetic sequence	a_n+1_ = a_n_+b_n_, b_n+1_ = b_n_+k	3, 4, 7, 12, 19
12	Arithmetic sequence	Geometric sequence	a_n+1_ = a_n_+b_n_, b_n+1_ = b_n_ × k	2, 3, 5, 9, 17
13	Arithmetic sequence	Addition sequence	a_n+1_ = a_n_+b_n_, b_n+2_ = b_n+1_+b_n_	3, 4, 6, 9, 14
14	Arithmetic sequence	Multiplication sequence	a_n+1_ = a_n_+b_n_, b_n+2_ = b_n+1_× b_n_	2, 3, 5, 7, 11
15	Geometric sequence	Arithmetic sequence	a_n+1_ = a_n_× b_n_, b_n+1_ = b_n_+k	1, 2, 8, 48, 384
16	Geometric sequence	Geometric sequence	a_n+1_ = a_n_× b_n_, b_n+1_ = b_n_× k	2, 2, 4, 16, 128
17	Geometric sequence	Addition sequence	a_n+1_ = a_n_× b_n_, b_n+2_ = b_n+1_+b_n_	3, 3, 6, 18, 90
18	Geometric sequence	Multiplication sequence	a_n+1_ = a_n_× b_n_, b_n+2_ = b_n+1_× b_n_	1, 2, 4, 16,128

*^∗^k is a constant. In the current study, k was an integer no larger than 5. a_n_ and a_n+1_ were consecutive elements in the primary number series, whereas b_n_, b_n+1_, and b_n+2_ were consecutive elements in the derivative number series, which were formed by increments or ratios derived from the primary number series.*

### Procedures and Materials

During item generation, in order to avoid unnecessary load incurred by arithmetic operation, we limited all starting numbers, increments and ratios (as well as numerators and denominators in fractions and numbers within a square root in irrational numbers) to a maximum value of 5. All number series had five number elements, with a periodicity of 1.

Six items were generated for each item model requiring one arithmetic operation (S1-S60). Four items were derived for each item model requiring two arithmetic operations (D1-D32). The resulting 92 items were divided into four item sets, each containing a number of anchor items so that all item sets could be linked (see Table 3 for details). Item sets 1 and 2 contained 35 items requiring one arithmetic operation, whereas the other two sets contained 44 items of requiring both one and two arithmetic operations.

**Table 3:** Description of the four item sets.

Item set	Unique items	Anchor items	Anchor items	Anchor items	Anchor items	Anchor items
1	S11-S15	S1-S10		S16-S35		
2	S46-S50			S16-S35	S36-S45	
3	D1-D8	S1-S10	S51-S60			D9-D24
4	D25-D32		S51-S60		S36-S45	D9-D24

*Items staring with S indicate items requiring one arithmetic operation, whereas items starting with D indicate items requiring two arithmetic operations.*

After completing the number series reasoning items, test takers were asked to answer five matrices reasoning problems selected from Raven’s Standard Progressive Matrices (Raven et al., 1988; Zhang and Wang, 1989) – one of the most commonly used measures of reasoning ability. The test includes five sets of matrices, each containing 12 items arranged according to difficulty. Only the 10th item in each set was used in the current study so as to shorten the test while maintaining a wide range of difficulty.

### Sample

To ensure that the sample resembled the target audience of the item generator, we reached out to a varied student population and selected a mixed group of participants with diverse educational backgrounds. A total of 211 participants were recruited from a vocational high school and an evening college in Beijing. Of these, 21.6% were male, and 78.4% were female. The average age was 22.54 years (*SD* = 6.05). A further 255 university students were also recruited. Of these, 21.2% were male, and 78.8% were female. The average age was 19.91 years (*SD* = 1.30). Item sets 3 and 4 were expected to be more difficult than item sets 1 and 2 due to the inclusion of items requiring both one and two arithmetic operations. Hence, university students were randomly assigned to item sets 3 or 4, whereas the vocational and evening college students were randomly assigned to item sets 1 or 2.

All participants received the paper-and-pencil test in the classroom and were informed that arithmetic equations were permissible answers (e.g., 3^∗^3^∗^3 instead of 27), in order to reduce the load on arithmetic operations. All participants under 16 years old have parental permission to participate in the test. All participants over the age of 16 volunteered to take part. Written informed consent was obtained from all participants over the age of 16 and from the parents/legal guardians of all participants below the age of 16.

## Results

Seven items were excluded from the final analysis. Six showed extremely high accuracy rates (>0.95), indicating a lack of discrimination. The seventh item (number series 2, 5, 7, 12, 19) was supposed to require two steps of arithmetic operation, but turned out to require only one; hence, it was removed from the analysis.

### Reliability

Data from the four item sets were analyzed with the Rasch model, using the software Conquest 2.0 (Wu et al., 2007). Concurrent equating was achieved given the presence of sufficient anchor items among the four sets. The results indicated good fit with the Rasch model, as most items (except three) showed acceptable infit and outfit indices between 0.8 and 1.4 (Linacre and Wright, 1994). Separation reliability was 0.98, whereas EAP/PV reliability was 0.87, suggesting satisfying reliability (Adams, 2005; Wu et al., 2007).

The Rasch model assumes the local independence of items. Violations of local independence can lead to inflated estimates of reliability and undermine construct validity. In order to check for local independence, we calculated Q_3_ fit statistics (Yen, 1984), which are the most widely used indicators of local dependence. Items generated from the same item model were used as testlets. Average *Q*_3_-values within and between testlets were 0.128 and 0.028, respectively; as these were both smaller than the critical value of 0.2 (Chen and Thissen, 1997; Makransky et al., 2014; Finch and Jeffers, 2016), we concluded that there was negligible dependence between items from the same item models and between items from different item models. Therefore, we were convinced that the assumption of the local independence of items was not violated.

### Validity

Five items from Raven’s Progressive Matrices were included in the current study. Cronbach’s alpha for these items was 0.612. The correlation coefficient between the EAP ability estimates from the Rasch model of the number series items and the raw scores of the Raven items, after correcting for attenuation, was 0.70 (*p* < 0.001), suggesting acceptable construct validity. A moderate correlation was expected, as the two tests–although both measuring the reasoning ability–differed in stimuli format (figures and numbers, respectively). Therefore, it was not surprising to observe that only 49% of variance (0.70^∗^0.70) was explained between the two tests.

### Estimation of Item Difficulty

#### Differences in Item Difficulty Between Items Requiring One and Two Arithmetic Operations

Item difficulty parameters were estimated using the Rasch model. The results revealed a significant difference in item difficulty between items requiring one and two arithmetic operations, *t*(83) = −6.29, *p* < 0.001. Items requiring two steps of arithmetic operation (average difficulty 1.28) were more difficult than those requiring one step (average difficulty −0.70).

#### Difficulty Levels of Items Requiring One Step of Arithmetic Operation

A two-way ANOVA (type of number element ∗ type of arithmetic operation) was conducted on the difficulty estimates of items requiring one arithmetic operation. The results showed that the main effect of the type of number element was significant, *F*(2,45) = 51.21, *p* < 0.001; the main effect of the type of arithmetic operation was significant, *F*(3,45) = 7.15, *p* < 0.01; and the interaction between the two variables was also significant, *F*(4,45) = 4.66, *p* < 0.01. All three effects accounted for 74.7% of the variance. *Post hoc* analysis with Bonferroni correction revealed significant differences in difficulty level between arithmetic sequences with integers and arithmetic sequences with fractions, *t*(45) = 7.18, *p* < 0.001; between addition sequences with integers and addition sequences with fractions, *t*(45) = 4.08, *p* < 0.001; between geometric sequences with integers and geometric sequences with square roots, *t*(45) = 5.48, *p* < 0.001; and between multiplication sequences with integers and multiplication sequences with square roots, *t*(45) = 4.35, *p* < 0.001. None of the remaining comparisons was significant.

**FIGURE 1:**
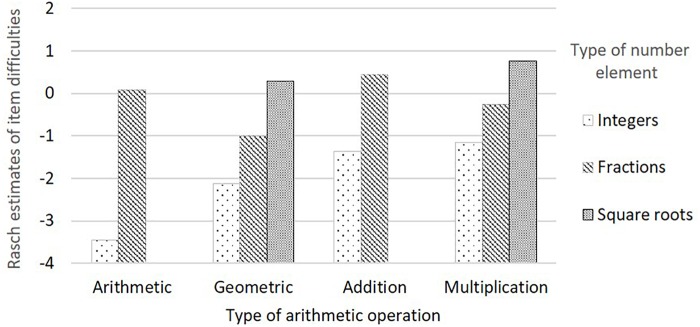
Rasch model estimates of item difficulties by type of arithmetic operation and type of number element.

Notably, Figure 1 reveals that number series with integers and number series with fractions displayed distinctive patterns across different arithmetic operations. It was more challenging for test takers to solve geometric sequences with integers than to solve arithmetic sequences with integers. The pattern with fractions was completely opposite. Our explanation for this is that fractions must be reduced to a common denominator before they can be added, whereas fractions can be multiplied by simply multiplying nominators and denominators, respectively. Therefore, it is more difficult to solve arithmetic sequences with fractions than to solve geometric sequences.

#### Difficulty Levels of Items Requiring Two Steps of Arithmetic Operation

Another two-way ANOVA analysis (type of first arithmetic operation ^∗^ type of second arithmetic operation) was conducted on items requiring two steps of arithmetic operation. The main effect of the type of the first arithmetic operation was significant, *F*(1,22) = 4.97, *p* < 0.05, showing that geometric sequences were more difficult to solve than arithmetic sequences (with average difficulties of 1.60 and 0.91, respectively). The main effect of the type of the second arithmetic operation was also significant, *F*(3,22) = 15.71, *p* < 0.001, with average difficulties of −0.09, 0.67, 1.95, and 2.58 for arithmetic, geometric, addition, and multiplication sequences, respectively. *Post hoc* analysis with Bonferroni correction showed that arithmetic sequences were significantly easier than addition and multiplication sequences, *t*(22) = −4.621, *p* < 0.01; *t*(22) = −6.253, *p* < 0.001, and geometric sequences were significantly less difficult than multiplication sequences, *t*(22) = −4.318, *p* < 0.01. The interaction between the two was not significant, *F*(3,22) = 0.347, *p* = 0.792. The two main effects accounted for 70.6% of the variance.

**Table 4:** Q-matrix of stimulus features.

Item model	GS	AS	MS	AS2	GS2	F	SR	GS^∗^F	AS^∗^F	MS^∗^F
1	0	0	0	0	0	0	0	0	0	0
2	1	0	0	0	0	0	0	0	0	0
3	0	1	0	0	0	0	0	0	0	0
4	0	0	1	0	0	0	0	0	0	0
5	0	0	0	0	0	1	0	0	0	0
6	1	0	0	0	0	1	0	1	0	0
7	0	1	0	0	0	1	0	0	1	0
8	0	0	1	0	0	1	0	0	0	1
9	1	0	0	0	0	0	1	0	0	0
10	0	0	1	0	0	0	1	0	0	0
11	0	0	0	1	0	0	0	0	0	0
12	1	0	0	1	0	0	0	0	0	0
13	0	1	0	1	0	0	0	0	0	0
14	0	0	1	1	0	0	0	0	0	0
15	0	0	0	0	1	0	0	0	0	0
16	1	0	0	0	1	0	0	0	0	0
17	0	1	0	0	1	0	0	0	0	0
18	0	0	1	0	1	0	0	0	0	0

*GS, geometric sequence; AS, addition sequence; MS, multiplication sequence; AS2, second arithmetic sequence; GS2, second geometric sequence; F, fraction numbers; SR, square roots.*

### Prediction of Item Difficulty From Stimulus Features

A Q-matrix was essential for the LLTM model to estimate the difficulty of stimulus features. In Table 4, each column presents one stimulus feature (i.e., rule). 1s and 0s indicate the presence or absence of corresponding stimulus features, respectively. There were 10 stimulus features in total, including 3 arithmetic operations, 2 additional arithmetic operations in the case of items requiring two steps of arithmetic operation, 2 number elements, and 3 interactions between fraction numbers and different arithmetic operations (as suggested in the ANOVA analysis 3.3.2). As the interaction between the two steps of arithmetic operation in items requiring two operations was not significant (indicated in the ANOVA analysis 3.3.3), such interaction terms were not included.

The Q-matrix below includes 18 item models, among which 10 models required one step of arithmetic operation and the remaining 8 required two steps. Arithmetic operations at the second step were exactly the same as those in items requiring one step; hence, both types could be modeled in the same Q-matrix, allowing for concurrent estimation with the LLTM. Arithmetic sequences with integers were considered a baseline, so *arithmetic sequence* and *integer* were not included in the Q-matrix. In the table, arithmetic sequences with integers (item model 1) are represented by the row with 0s in all cells.

**Table 5:** Difficulty levels of stimulus features.

Stimulus feature	Estimate	*SE*	*t*
Geometric sequence	0.98	0.12	8.17^∗∗∗^
Addition sequence	1.77	0.12	14.75^∗∗∗^
Multiplication sequence	2.12	0.10	21.20^∗∗∗^
Second arithmetic sequence	3.81	0.10	38.10^∗∗∗^
Second geometric sequence	4.26	0.09	47.33^∗∗∗^
Fractions	3.15	0.13	24.23^∗∗∗^
Square roots	2.22	0.09	24.67^∗∗∗^
Geometric ∗ fractions	−1.78	0.16	−11.13^∗∗∗^
Addition ∗ fractions	−1.35	0.15	−9.00^∗∗∗^
Multiplication ∗ fractions	−2.33	0.15	−15.53^∗∗∗^

*^∗∗∗^p < 0.001.*

Based on the Q-matrix, the difficulties of stimulus features were estimated by the LLTM. As shown in Table 5, the results were consistent with our previous ANOVA analyses. Specifically, items with fractions and square roots were more difficult than items with integers. Arithmetic sequences, geometric sequences, addition sequences, and multiplication sequences were successively more difficult. A second arithmetic sequence was easier than a second geometric sequence. The imposition of a second arithmetic operation introduced more difficulty to an item. All values were relative to the baseline level of items of a single arithmetic sequence with integers.

**Table 6:** Difficulty levels estimated from stimulus features and the Rasch model.

Item model	Type of number element	Number of arithmetic operations	Type(s) of arithmetic operation	Difficulty by LLTM	Difficulty by Rasch (averaged within the item model)
1	Integer	1	Arithmetic sequence	0	−3.46
2	Integer	1	Geometric sequence	0.98	−2.12
3	Integer	1	Addition sequence	1.77	−1.36
4	Integer	1	Multiplication sequence	2.12	−1.16
5	Fraction	1	Arithmetic sequence	3.15	0.11
6	Fraction	1	Geometric sequence	2.35	−1.00
7	Fraction	1	Addition sequence	3.56	0.44
8	Fraction	1	Multiplication sequence	2.94	−0.23
9	Square root	1	Geometric sequence	3.20	0.29
10	Square root	1	Multiplication sequence	4.34	0.76
11	Integer	2	Arithmetic sequence + Arithmetic sequence	3.81	−0.47
12	Integer	2	Arithmetic sequence + Geometric sequence	4.79	0.04
13	Integer	2	Arithmetic sequence + Addition sequence	5.58	1.53
14	Integer	2	Arithmetic sequence + Multiplication sequence	5.93	2.48
15	Integer	2	Geometric sequence + Arithmetic sequence	4.26	0.29
16	Integer	2	Geometric sequence + Geometric sequence	5.24	1.14
17	Integer	2	Geometric sequence + Addition sequence	6.03	2.27
18	Integer	2	Geometric sequence + Multiplication sequence	6.38	2.69

**FIGURE 2:**
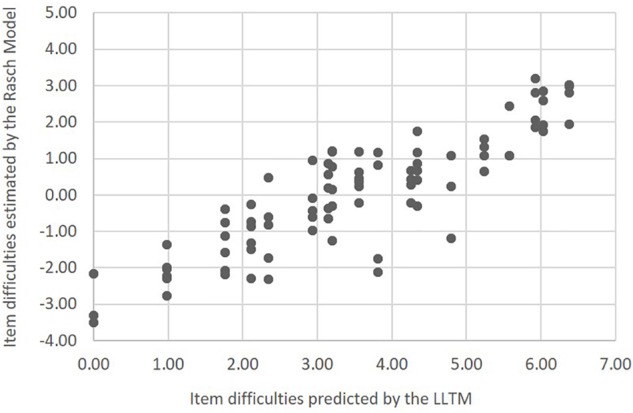
Scatter plot of item difficulties predicted by the LLTM by Rasch model estimates.

Based on Table 5, we were able to estimate the difficulty levels of all items by summing the difficulty estimates of corresponding stimulus features. For instance, the difficulty of an integer item with an arithmetic sequence at step 1 and an addition sequence at step 2 was 5.58 (3.81+1.77); and the difficulty of a fraction item with a geometric sequence was 2.35 [0.98 + 3.15 + (−1.78)]. Using the LLTM estimation results, the difficulty levels of all items created by the item generator were predicted easily. Table 6 presents the item difficulties derived from the LLTM and those estimated directly by the Rasch model for each item model. Item-level difficulty estimates are available in the Supplementary Appendix. Although the two estimations were on different scales, Figure 2 shows that there was a high correlation between the two sets (*r* = 0.97 on the item model level and *r* = 0.88 on the item level, both *p*s < 0.001). In another words, 77.4% of the variance in item difficulty (0.88^∗^0.88) was explained by the stimulus features implemented in the automatic item generator. This suggests that the automatic item generator was capable of generating items with highly predictable item difficulty. More importantly, the items created by the item generator covered a wide range of item difficulty levels (from −3.46 to 2.69), indicating the potential to generate items of high difficulty.

## Discussion

The current study successfully developed an automatic item generator for the number series completion problem built on a new set of stimulus features. While controlling for irrelevant features, the generator proved capable of creating items of a wide range of difficulty levels–particularly those of high difficulty levels. Results from the LLTM analysis showed significant contributions of stimulus features to the difficulty levels of resulting items. High correlation was observed between the ability estimates from the Rasch model and the LLTM, suggesting strong predictability of item difficulty. With increased efficiency in developing new items of predictable difficulty, the generator will benefit future test development relating to numerical reasoning.

The current study investigated three classes of stimulus features: type of number element, type of arithmetic operation, and number of arithmetic operations. In addition to integers, fractions and square roots were introduced into the number series reasoning items, and this effectively increased item difficulty. Four types of arithmetic operations were adopted: arithmetic, geometric, addition, and multiplication sequences. These different arithmetic operations exhibited varying difficulty levels, probably due to inconsistent levels of familiarity as a result of previous training. In an attempt to further elevate item difficulty, we created items that required participants to carry out two steps of arithmetic operation. As expected, these items were significantly more difficult than those requiring one arithmetic operation. Covering a broad range of difficulty, the items derived from the generator accommodated both junior and advanced test takers with variable ability levels.

The new item generator differed from the one developed by Arendasy and Sommer (2012), as it paid more attention to the essential elements of the number series than the cognitive framework. Acknowledging the impact of stimulus features on mental processes and working memory, the current study clarified the terms of rule span, number of rules, and rule complexity by proposing three classes of stimulus features that elucidated the rules of the number series completion problem (i.e., what are the numbers, how are they related, and how do these two interact). It turned out that such rules were capable of predicting item difficulty with considerably high accuracy. More importantly, this approach greatly benefited the test construction. Item difficulty could be easily predicted when items were constructed, as all elements were included in the LLTM model. Following the principle of assessment engineering (Luecht, 2013), rule-based item models were considered expandable. When more rules were introduced into the LLTM, item difficulties could be predicted with a simple linear calculation.

Another strength of the new item generator was its capacity to generate items of high difficulty. It is not common for number series reasoning items to adopt fractions and irrational numbers. As shown in the results, fractions imposed an additional requirement for participants, particularly in items requiring addition. The presence of square roots also substantially increased item difficulty, as test takers were required to understand the concept of square roots in order to solve the problem. The generator incorporated four types of arithmetic operation, yielding eight different item models requiring two steps of arithmetic operation. Participants had to not only identify relations among adjacent numbers, but also maintain increments or ratios in working memory and deduct a second-order relation that could be the same or different from the initial one. This complex process of completing a number series with only five numbers proved extremely difficult.

Notably, the study carefully constrained irrelevant features. For example, it has been suggested that cognitive load resulting from pure arithmetic operation is not relevant to reasoning ability (Arendasy and Sommer, 2012). Thus, in our study, we limited the starting numbers, increments, and ratios to values below 5 so as to minimize the interference of arithmetic operation. We also accepted formulae as answers, in order to reduce unnecessary errors in arithmetic operation. In comparison to other automatic item generators, our generator proved effective in generating items of predictable item difficulty (with 77.4% of variance explained) with a wide range of difficulty levels (above 6 theta units from −3.46 to 2.69).

Nonetheless, it is noted that the items within the same item models still exhibited variable difficulty levels, although they were designed following the same rules. Future studies are warranted to search for additional features that account for the currently unexplained variance in item difficulty. For such purpose, more items have to be generated, which would require an even larger sample. The current study was restrained by the relatively small sample size. It is also important to replicate the study in a different, larger and more heterogeneous sample, preferably a representative one, if the generator is going to be applied in high-stake testing scenarios.

Moreover, the item generator was limited by the setting of a periodicity of 1, as only one number series with five numbers was present in each item. On the one hand, this helped ensure that the number series were all of the same length. Had there been more than one number series, the number of elements would have been reduced, and this might have introduced confounding variables. On the other hand, this practice prevented the generator from creating items of even more difficult items including more than one number series. In future research, we plan to broaden the scope of item generation, expanding the periodicity to more than 1. Additionally, it would be interesting to observe item difficulty with larger variance on other features by, for instance, incorporating more arithmetic operations (such as power and factorial operations), including negative numbers, and requiring more than two steps of arithmetic operation.

## Conclusion

Number series reasoning items are extensively used in educational assessment. In daily practice, test developers are primarily concerned with the quality of the items. While reliability and validity evidence is frequently reported, the exact range of item difficulty levels is not always available, although this is indeed pertinent to the reliability and validity of a test. As automatic item generation becomes increasingly popular, more attention is being paid to underlying cognitive processes and the predictive power of the rules in the item generator. This article described the design of a new number series reasoning item generator that catered the need for more challenging items. Specific stimulus features that contributed to a high difficulty level were incorporated. On the basis of rule-based item models, we not only demonstrated that item difficulty could be effectively predicted from the stimulus features, but we also ensured that the resulting items covered a broad range of difficulty levels that is required for test administration in specific contexts. We hope that our unique approach is inspiring to test developers and can be applied to the development of other reasoning item types.

## Ethics Statement

This study was carried out in accordance with the principles of voluntary basis, confidentiality and security recommended by the Ethics and Human Safety Commission, School of Psychology, Beijing Normal University. The protocol was approved by the Ethics and Human Safety Commission, School of Psychology, Beijing Normal University.

## Author Contributions

FL designed the experiments, supervised the research process, and provided the financial support. YL gathered the data. LS analyzed the data and wrote the manuscript.

## Conflict of Interest Statement

The authors declare that the research was conducted in the absence of any commercial or financial relationships that could be construed as a potential conflict of interest.
