# Supplementary material for: Automatic Generation of Number Series Reasoning Items of High Difficulty
Source: Front Psychol. 2019 Apr 24;10:884. doi: 10.3389/fpsyg.2019.00884 (PMC6491774; doi:10.3389/fpsyg.2019.00884)
Supplement: Supplementary file 1 [file Table_1.DOCX]

Appendix. Item difficulties estimated by the Rasch model and those predicted by the LLTM

| Item | Item Model | Item difficulty estimated by the Rasch model | Item difficulty predicted by the LLTM |
| --- | --- | --- | --- |
| 1 | 1 | -4.88 | 0.00 |
| 2 | 1 | -3.49 | 0.00 |
| 3 | 1 | -3.30 | 0.00 |
| 4 | 1 | -2.17 | 0.00 |
| 5 | 2 | -2.78 | 0.98 |
| 6 | 2 | -2.30 | 0.98 |
| 7 | 2 | -2.23 | 0.98 |
| 8 | 2 | -2.04 | 0.98 |
| 9 | 2 | -1.99 | 0.98 |
| 10 | 2 | -1.36 | 0.98 |
| 11 | 3 | -2.18 | 1.77 |
| 12 | 3 | -2.09 | 1.77 |
| 13 | 3 | -1.58 | 1.77 |
| 14 | 3 | -1.13 | 1.77 |
| 15 | 3 | -0.77 | 1.77 |
| 16 | 3 | -0.40 | 1.77 |
| 17 | 4 | -2.29 | 2.12 |
| 18 | 4 | -1.49 | 2.12 |
| 19 | 4 | -1.33 | 2.12 |
| 20 | 4 | -0.86 | 2.12 |
| 21 | 4 | -0.75 | 2.12 |
| 22 | 4 | -0.25 | 2.12 |
| 23 | 5 | -0.65 | 3.15 |
| 24 | 5 | -0.37 | 3.15 |
| 25 | 5 | 0.18 | 3.15 |
| 26 | 5 | 0.55 | 3.15 |
| 27 | 5 | 0.86 | 3.15 |
| 28 | 6 | -2.31 | 2.35 |
| 29 | 6 | -1.72 | 2.35 |
| 30 | 6 | -0.82 | 2.35 |
| 31 | 6 | -0.61 | 2.35 |
| 32 | 6 | 0.47 | 2.35 |
| 33 | 7 | -0.22 | 3.56 |
| 34 | 7 | 0.23 | 3.56 |
| 35 | 7 | 0.36 | 3.56 |
| 36 | 7 | 0.46 | 3.56 |
| 37 | 7 | 0.62 | 3.56 |
| 38 | 7 | 1.18 | 3.56 |
| 39 | 8 | -0.97 | 2.94 |
| 40 | 8 | -0.60 | 2.94 |
| 41 | 8 | -0.43 | 2.94 |
| 42 | 8 | -0.08 | 2.94 |
| 43 | 8 | 0.94 | 2.94 |
| 44 | 9 | -1.26 | 3.20 |
| 45 | 9 | -0.31 | 3.20 |
| 46 | 9 | 0.16 | 3.20 |
| 47 | 9 | 0.78 | 3.20 |
| 48 | 9 | 1.19 | 3.20 |
| 49 | 9 | 1.21 | 3.20 |
| 50 | 10 | -0.30 | 4.34 |
| 51 | 10 | 0.41 | 4.34 |
| 52 | 10 | 0.67 | 4.34 |
| 53 | 10 | 0.85 | 4.34 |
| 54 | 10 | 1.16 | 4.34 |
| 55 | 10 | 1.76 | 4.34 |
| 56 | 11 | -2.11 | 3.81 |
| 57 | 11 | -1.74 | 3.81 |
| 58 | 11 | 0.81 | 3.81 |
| 59 | 11 | 1.16 | 3.81 |
| 60 | 12 | -1.19 | 4.79 |
| 61 | 12 | 0.24 | 4.79 |
| 62 | 12 | 1.08 | 4.79 |
| 63 | 13 | 1.08 | 5.58 |
| 64 | 13 | 1.08 | 5.58 |
| 65 | 13 | 2.44 | 5.58 |
| 66 | 14 | 1.86 | 5.93 |
| 67 | 14 | 2.06 | 5.93 |
| 68 | 14 | 2.79 | 5.93 |
| 69 | 14 | 3.20 | 5.93 |
| 70 | 15 | -0.22 | 4.26 |
| 71 | 15 | 0.28 | 4.26 |
| 72 | 15 | 0.43 | 4.26 |
| 73 | 15 | 0.66 | 4.26 |
| 74 | 16 | 0.65 | 5.24 |
| 75 | 16 | 1.07 | 5.24 |
| 76 | 16 | 1.32 | 5.24 |
| 77 | 16 | 1.53 | 5.24 |
| 78 | 17 | 1.74 | 6.03 |
| 79 | 17 | 1.92 | 6.03 |
| 80 | 17 | 2.58 | 6.03 |
| 81 | 17 | 2.84 | 6.03 |
| 82 | 18 | 1.95 | 6.38 |
| 83 | 18 | 2.81 | 6.38 |
| 84 | 18 | 2.98 | 6.38 |
| 85 | 18 | 3.01 | 6.38 |
